# AS-UNet: A Lightweight U-Net with Asymmetric Strip Attention and Joint Gating for RGB Optical Landslide Segmentation

**DOI:** 10.3390/s26154820

**Published:** 2026-07-29

**Authors:** Haoran You, Cong Wang, Yunbai Qin, Hua Wu, Zheli Tang, Zhuoxiang Lin

**Affiliations:** School of Electronic and Information Engineering, Guangxi Normal University, Guilin 541004, China; youhaoran@stu.gxnu.edu.cn (H.Y.); cong@stu.gxnu.edu.cn (C.W.); hwu@stu.gxnu.edu.cn (H.W.); tzl@stu.gxnu.edu.cn (Z.T.); linzx@stu.gxnu.edu.cn (Z.L.)

**Keywords:** landslide segmentation, RGB optical imagery, remote sensing, semantic segmentation, lightweight network

## Abstract

Accurate delineation of landslides in RGB optical remote sensing imagery supports rapid disaster mapping and post-event assessment. This remains difficult because landslides are often small and irregular, resemble bare soil or disturbed vegetation, and acquire blurred boundaries when images are resized. We developed AS-UNet, a lightweight U-Net variant with three targeted modifications. The Asymmetric Strip Attention Module uses horizontal and vertical depthwise strip convolutions with parallel channel-spatial reweighting to capture anisotropic landslide morphology. The Channel-Spatial Joint Gate uses decoder semantics to filter selected skip connections while retaining channel-specific spatial responses. The Poly-Harmonized Gradient Dice Loss (PGD Loss) combines pixel-wise, region-overlap, gradient-density, and probability-regularization terms for imbalanced segmentation. At 128 × 128 input resolution, AS-UNet achieved a best-validation IoU of 80.46 ± 0.03% and an independent-test IoU of 77.82 ± 0.32% across three random seeds. AS-UNet contains 8.634 M parameters and processed 380.79 frames per second on the reported hardware. These results indicate a favorable balance between segmentation accuracy and computational efficiency for RGB optical landslide mapping.

## 1. Introduction

Landslides are destructive geological hazards that can cause casualties, infrastructure damage, and long-term disruption of land use [1,2]. Rapid and accurate delineation of affected areas is therefore important for emergency response, damage assessment, and geological-hazard monitoring [3]. These operational needs make timely and repeatable mapping over large areas a practical requirement.

Remote sensing provides a practical basis for meeting this requirement because it enables repeated observation of large or inaccessible regions. It has consequently become a central source of evidence for landslide inventory mapping. Conventional workflows rely on expert visual interpretation or classification rules derived from spectral, textural, and topographic features [3]. Although interpretable, these approaches are labor-intensive and transfer poorly when thresholds or hand-crafted features are tied to a particular scene. Automated mapping must therefore reduce dependence on manual rules while remaining responsive to scene-specific landslide patterns.

The design of such a mapping system depends first on the sensing modality. Landslide mapping and monitoring can use RGB optical imagery, synthetic aperture radar (SAR), digital elevation models, and multispectral data. Optical images provide intuitive color, texture, and morphological information, whereas SAR offers day–night and all-weather acquisition. SAR-based studies have addressed failure mapping and rapid detection [4,5], while interferometric observations support displacement monitoring [6,7]. Because these modalities have different imaging mechanisms and degradation sources, they should not be treated as interchangeable inputs. Accordingly, the present study is restricted to three-channel RGB optical image–mask pairs; SAR, DEM-assisted, multispectral, and multimodal fusion are outside the evaluated scope.

Within this defined RGB setting, remote-sensing research is expanding from task-specific deep networks toward foundation models [8], while landslide-specific studies document substantial progress in automated identification [9]. Fully convolutional networks and U-Net-based encoder–decoder architectures enable end-to-end pixel-level prediction and multi-scale feature fusion [10,11]. Replacing hand-crafted rules with an end-to-end network, however, does not remove the main image-level difficulties. Three issues are especially relevant here: sparse landslide foreground pixels create severe class imbalance; elongated or irregular landslide bodies are poorly matched by exclusively square receptive fields; and shallow features from spectrally similar backgrounds can pass through skip connections and generate false positives. Resizing heterogeneous source images to a common network input can further weaken local texture and boundary cues.

These coupled challenges have motivated several complementary lines of work. General channel-spatial attention and self-attention improve feature selection or long-range context [12,13], while strip pooling enlarges the receptive field along individual directions [14]. Cost-sensitive cross-entropy, Focal Loss, Lovasz-Softmax, Dice Loss, boundary-aware objectives, gradient harmonization, and polynomial probability modulation address different aspects of imbalance or boundary optimization [15,16,17,18,19,20,21,22]. Each line targets one part of the problem. They have not, however, been jointly tailored to anisotropic landslide morphology, background-contaminated skip connections, and hard-pixel optimization within the same lightweight RGB segmentation framework.

This combination of unresolved issues motivated the creation of AS-UNet, a lightweight U-Net variant for RGB optical landslide segmentation. Rather than treating the three difficulties separately, the model couples directional feature extraction at the bottleneck with channel-preserving skip-connection gating and an imbalance-aware composite objective. The contributions are as follows:

(1) We introduce an Asymmetric Strip Attention Module (ASAM) that uses 3 × 7 and 7 × 3 depthwise strip-convolution branches, an identity branch, and parallel channel-spatial reweighting. This design expands anisotropic context while retaining a compact path for locally bounded landslide regions.
(2) We introduce a Channel-Spatial Joint Gate (CSJG) in selected skip connections. Decoder semantics guide the filtering of encoder features without collapsing the attention map into a single spatial channel, thereby limiting irrelevant shallow responses while retaining channel-specific spatial weights.
(3) We propose the Poly-Harmonized Gradient Dice Loss (PGD Loss), which combines binary cross-entropy and image-wise Dice supervision with a PolyGHM term integrating gradient-density reweighting and first-order probability regularization. This design prevents abundant easy background pixels from dominating optimization, increases the contribution of sparse difficult pixels, and retains direct supervision of region overlap under severe foreground-background imbalance.

## 2. Related Work

### 2.1. Remote Sensing Modalities for Landslide Mapping

Remote-sensing landslide mapping has progressed from rule-based image interpretation toward object-based learning. Early workflows combined visual interpretation with spectral thresholds, vegetation indices, texture measures, and topographic variables. Object-Based Image Analysis (OBIA) subsequently introduced spatial objects and classifiers such as support vector machines and random forests [23,24,25]. These methods improve spatial consistency but remain sensitive to segmentation scale, feature selection, and regional calibration. This dependence limits transfer across landscapes and acquisition conditions and helps explain the later shift toward end-to-end representation learning.

Before considering that shift, the distinction between optical and radar observation is important. SAR complements optical observation because it can acquire data through cloud cover and without daylight. Amplitude, coherence, and learning-based SAR products have been used for landslide-failure mapping and rapid detection [4,5], whereas MTInSAR and DInSAR products quantify surface displacement [6,7]. SAR therefore belongs in the broader monitoring context, but it is not interchangeable with RGB optical imagery. Speckle, geometric distortion, phase decorrelation, and viewing geometry differ from the illumination, shadow, color, and texture limitations of optical data. These differences define the scope of the subsequent method comparison: AS-UNet is evaluated only on RGB optical imagery, with no claim of cross-modality performance.

### 2.2. Deep Learning for Landslide Segmentation

Within the RGB optical scope defined above, deep segmentation replaces hand-crafted classification pipelines with end-to-end feature learning. FCN [10] and U-Net [11] established pixel-level prediction and encoder–decoder fusion. Landslide4Sense subsequently provided a public multi-source remote-sensing benchmark together with reference deep-learning models for landslide detection [26]. DeepLab and PSPNet use atrous or pyramid context aggregation [27,28], while recent landslide-oriented studies have explored windowed Transformers, multiscale lightweight designs, and deep neural segmentation [29,30,31]. Despite this progress, RGB landslide scenes still combine anisotropic target morphology, foreground sparsity, and background textures that resemble exposed landslide material.

These remaining limitations are coupled rather than independent. Directional morphology affects contextual representation, confusing shallow textures affect skip fusion, and foreground sparsity affects optimization. AS-UNet therefore assigns a distinct component to each stage: ASAM models directional context at the bottleneck, CSJG filters shallow background responses during skip fusion, and PGD Loss addresses the imbalance between abundant easy pixels and sparse difficult foreground or boundary pixels. Section 4.4 evaluates the contribution of these components through ablation.

### 2.3. Attention and Strip-Structure Methods

The first design issue is how to represent elongated and irregular landslide context. Channel, spatial, and self-attention mechanisms can recalibrate feature responses or model long-range dependencies. DANet jointly models spatial and channel dependencies [12], while Vision Transformers extend context modeling through global self-attention [13]. Strip pooling instead uses elongated horizontal and vertical operators to capture long-range context with limited cost [14]. General attention is not explicitly aligned with landslide directionality, whereas a fixed strip operator alone cannot determine how much directional or identity information each feature map should retain.

ASAM is positioned at the intersection of these two lines of work. It combines two asymmetric depthwise strip-convolution branches with an identity branch and estimates channel and spatial branch weights in parallel. The strip branches provide directional context, while the identity path preserves compact local structure. Accordingly, the contribution lies in the task-oriented branch construction and joint reweighting strategy, not in the invention of attention or strip convolution themselves.

### 2.4. Skip-Connection Gating

The second design issue concerns how encoder detail is transferred to the decoder. U-Net skip connections restore fine spatial structure but can also transmit shallow responses from bare soil, roads, rocks, riverbanks, and vegetation. Attention U-Net addresses this problem by using decoder features to gate encoder features before fusion [32]. Its canonical gate produces a spatial attention map after channel projection, which supports localization but compresses channel-specific responses.

CSJG addresses this trade-off while retaining the decoder-guided gating principle. After a single projection for channel alignment, it produces channel-specific spatial weights rather than one shared spatial mask. The design is intended to reduce background transfer without discarding variation across feature channels. Its distinction from prior attention gates therefore lies in the retained channel dimension and the associated lightweight implementation.

### 2.5. Imbalance- and Boundary-Aware Objectives

The third design issue arises during optimization. Even with improved representation and fusion, sparse foregrounds and ambiguous boundaries can bias learning toward easy background pixels. Weighted cross-entropy [15], Focal Loss [16], Lovasz-Softmax [17], Dice Loss [18], Boundary Loss [19], and attention-boundary combinations [20] address different aspects of this problem. Gradient harmonization reweights samples according to gradient density [21], whereas PolyLoss modifies the probability expansion of cross-entropy [22]. These objective families are complementary, although fixed hard-sample weighting can also amplify noisy or ambiguous pixels.

PGD Loss is positioned as a composite response to these complementary objectives. It combines gradient-density reweighting, first-order probability regularization, binary cross-entropy, and image-wise Dice overlap. Within the reported binary RGB landslide setting, this combination jointly addresses easy-background dominance, difficult-pixel confidence, and region overlap. Section 3.4 retains the complete mathematical definition, while Section 4.6 reports the empirical μ sensitivity and representative loss-function trajectories.

Viewed together, prior work establishes a progression from sensing modality to contextual representation, feature transfer, and optimization. Within this progression, AS-UNet organizes three established lines of reasoning into explicitly positioned components. Their effects are assessed through ablation, repeated-seed comparisons, and efficiency measurements. The resulting evidence is limited to the evaluated RGB optical setting; multimodal and cross-dataset generalization remain outside the present experiments.

## 3. Method

This study develops AS-UNet from the U-Net encoder–decoder architecture for binary landslide segmentation in RGB optical imagery. This section describes the overall architecture, ASAM, CSJG, the unchanged PGD Loss formulation, and the activation function used in the implementation.

### 3.1. AS-UNet Architecture

**Figure 1:**
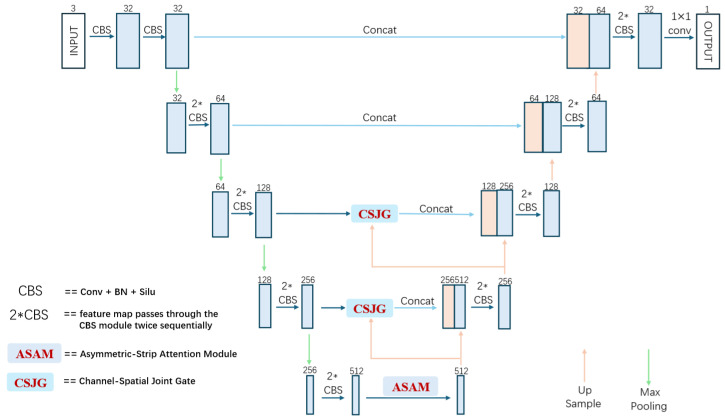
Overall architecture of AS-UNet.

AS-UNet comprises an encoder–decoder backbone, ASAM at the bottleneck, CSJG modules in selected skip connections, bilinear upsampling, and the final segmentation head. Figure 1 summarizes the data flow and the spatial scales used by the network.

The encoder progressively extracts multi-scale features, and the decoder restores spatial resolution through bilinear interpolation and fusion with gated encoder features. ASAM operates at the bottleneck to aggregate horizontal, vertical, and identity-path information. CSJG is applied to the third and fourth skip connections to reduce irrelevant shallow responses before concatenation. Orange arrows in Figure 1 denote bilinear upsampling and green arrows denote max-pooling downsampling.

### 3.2. Asymmetric Strip Attention Module

Jie Hu et al. first proposed the Squeeze-and-Excitation Networks (SENet) model to optimize feature representation in deep learning [33]. By illustrating the dependency relationships among global modeling channels, adaptive calibration of feature weights between channels has been achieved. However, although SENet establishes important relationships between channels, it ignores the spatial distribution patterns of feature information. To address this deficiency, Sanghyun Woo et al. innovatively adopted a serial topology structure and proposed the Convolutional Block Attention Module (CBAM) [34]. Connecting a spatial attention module after the channel attention mechanism enables dual-domain feature extraction. The forward propagation process proceeds as follows:

(1) $$M_{c}=\sigma (MLP\left(AvgPool\left(F\right)\right)+MLP(MaxPool(F)))$$

(2) $$F^{\prime }=F⨂M_{c}$$

(3) $$M_{s}=\sigma ({Conv}_{7\times 7}([AvgPool\left(F^{\prime }\right);MaxPool(F^{\prime })]))$$

(4) $$Output=M_{s}⨂F^{\prime }$$

where F is the input feature map; MLP is a multi-layer perceptron; σ is the sigmoid activation function; $M_{c}$ is the channel feature map; $M_{s}$ is the spatial feature map; and ⊗ represents element-wise multiplication under the broadcasting mechanism.

CBAM is lightweight and broadly applicable, but two aspects motivate the development of a task-specific alternative for RGB landslide segmentation.

First, the 7 × 7 spatial operator used in CBAM applies the same receptive-field extent in both directions. Landslide regions can instead extend preferentially along slopes, valleys, or river corridors; an anisotropic operator can capture such context with fewer kernel elements than an equally wide square convolution.

Second, CBAM estimates channel attention before spatial attention. This serial dependence can constrain the spatial weighting stage when informative directional responses occur in only a subset of feature channels. ASAM therefore uses directional branches and estimates channel and spatial branch weights in parallel.

On this basis, we introduce ASAM, which combines asymmetric strip convolution with parallel channel and spatial branch reweighting.

**Figure 2:**
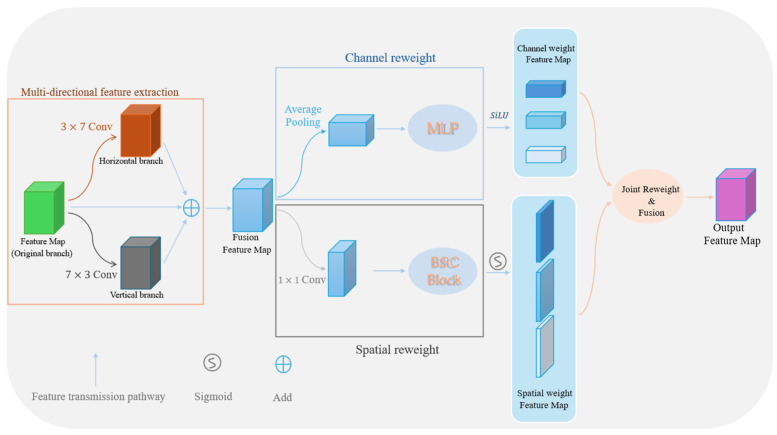
Architecture of the Asymmetric Strip Attention Module (ASAM).

As shown in Figure 2, ASAM contains three stages: multi-directional feature extraction, dual-domain dynamic weight generation, and joint feature fusion. The following subsections describe each stage.

#### 3.2.1. Multi-Directional Feature Extraction

Motivated by factorized convolution and strip pooling [14,35], ASAM replaces a single symmetric square operator with parallel horizontal, vertical, and identity paths to capture long-range directional context while retaining local information.

Assuming the input feature map is $X\in R^{N\times C\times H\times W}$, where N, C, H, and W represent the batch size, number of channels, height, and width, respectively, ASAM sends it to the horizontal feature branch, vertical feature branch, and identity mapping branch, respectively.

The horizontal feature branch employs a 3 × 7 strip convolution to capture the contextual features of landslides extending laterally. The output is denoted as $X_{h}\in R^{N\times C\times H\times W}$.

The vertical feature branch employs a 7 × 3 strip convolution to capture the contextual features of landslides extending longitudinally. The output is denoted as $X_{w}\in R^{N\times C\times H\times W}$.

The identity mapping branch directly preserves the original local spatial and fine texture features, producing an output denoted as X.

To control model complexity, the horizontal and vertical branches use depthwise grouped convolution [36]. Considering the strip branches alone, a standard 7 × 7 convolution contains 49C^2^ kernel parameters, whereas 3 × 7 and 7 × 3 depthwise convolutions together contain 42C kernel parameters. The remaining fusion and reweighting operations add parameters, but the directional branches expand the anisotropic receptive field without the quadratic channel cost of a standard 7 × 7 convolution.

#### 3.2.2. Dual-Domain Dynamic Weight Generation

To effectively screen and differentiate the features of the three aforementioned forms, ASAM introduces a dual-domain dynamic weight generation mechanism that operates in parallel across channels and spatial dimensions. Firstly, we add the features of the three branches element-wise to construct an aggregated feature that contains multi-directional global context $X_{add}$:

(5) $$X_{add}=X_{h}+X_{w}+X$$

Subsequently, $X_{add}$ is simultaneously fed into two subnetworks: Channel Reweight and Spatial Reweight.

**Figure 3:**
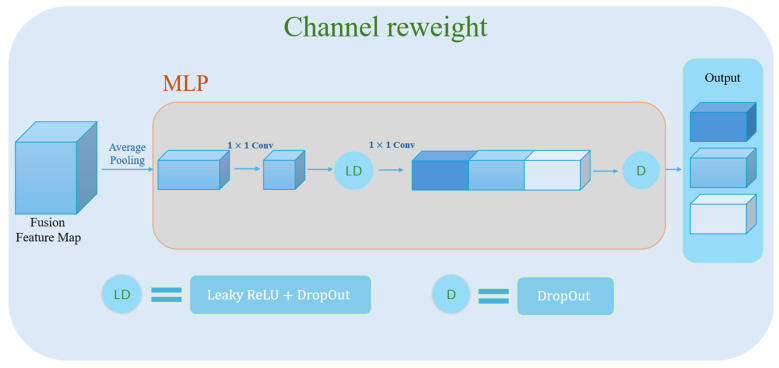
Channel-reweighting branch of ASAM. Global average pooling and a bottleneck MLP generate branch-specific channel weights for the horizontal, identity, and vertical features.

The channel reweighted network is illustrated in Figure 3. Due to the strong spectral or textural response of landslides in certain specific high-dimensional feature channels, this branch aims to adaptively calibrate the importance of each channel. Firstly, $X_{add}$ is compressed to an N × C × 1 × 1 spatial dimension through Global Average Pooling (GAP) and then fed into a Multi-Layer Perceptron (MLP). This MLP features a bottleneck structure with a dimensionality reduction ratio of 2, after which the output is upscaled to 3C and reshaped into channel feature maps with three independent branches. Finally, the feature channel map, reshaped by the channel reweighting network, is activated by the SiLU function to generate a channel weight matrix:

(6) $$A_{c}=SiLU(MLP\left(GAP\left(X_{add}\right)\right))$$

The reason for abandoning the traditional Sigmoid function and adopting the SiLU function here is that SiLU preserves smooth and small gradients in the negative value region. This feature allows the network to suppress redundant background channels without entirely eliminating neurons that represent weak landslide edge signals, thereby preserving richer feature representations. The self-gating property of SiLU enables the model to dynamically adjust response strength based on the local saliency of landslide targets, which is especially important for extracting weak signals in RGB optical imagery.

**Figure 4:**
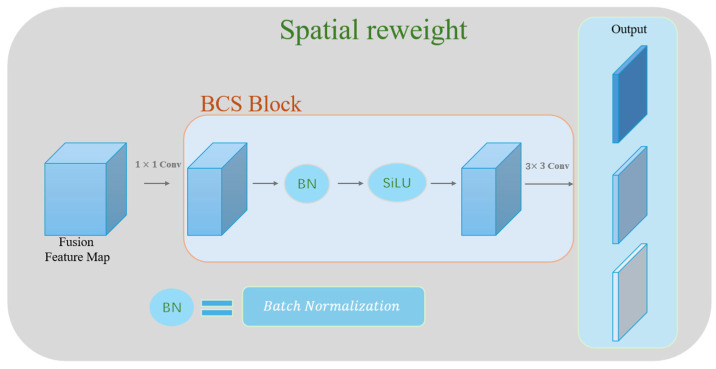
Spatial-reweighting branch of ASAM. Convolution, batch normalization, and SiLU activation generate branch-specific spatial weight maps from the aggregated feature.

The spatial reweighting network is illustrated in Figure 4. The complex background information of landslides, including exposed rocks and similar vegetation, demands that networks possess exceptionally high spatial recognition capabilities at the pixel level. This branch first applies a 1 × 1 convolution to reduce the dimensionality of $X_{add}$ to C/4, followed by batch normalization (BN) and SiLU activation. It then maps the features into a spatial feature map with three channels using a 3 × 3 convolution. Finally, the spatial feature map output by the spatial reweighting network is activated using the sigmoid function to generate a spatial weight matrix.

(7) $$A_{s}=\sigma ({Conv}_{3\times 3}(SiLU(BN({Conv}_{1\times 1}(X_{add})))))$$

#### 3.2.3. Joint Feature Fusion

After obtaining the channel and spatial weight matrices separately, ASAM abandons the sequential processing approach used in CBAM and instead employs a dynamic parallel processing strategy to jointly reconstruct features across multiple paths.

**Figure 5:**
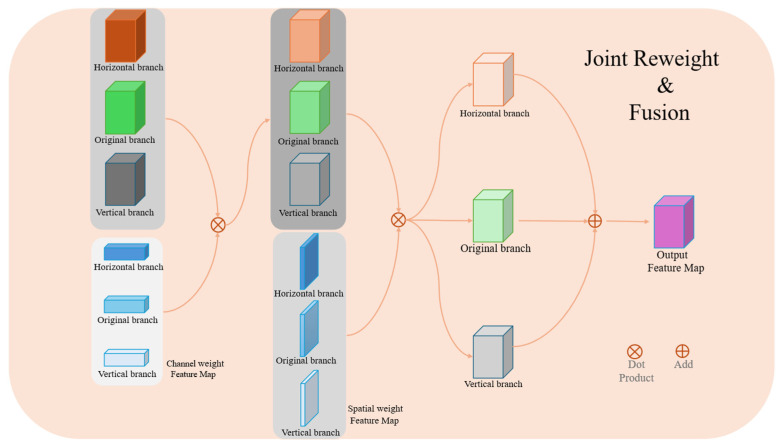
Joint reweighting and feature fusion in ASAM. The three feature branches are modulated by their channel and spatial weights and then summed to produce the output feature.

As shown in Figure 5, the network multiplies the three extracted feature branches ($X_{h}$, $X_{w}$, X) element-wise by their corresponding channel and spatial weights, then sums the weighted features to produce the final fused feature map, $X_{out}$. The mathematical expression is as follows:

(8) $$X_{out}=\sum_{i\in \{h,w,id\}}\left(X_{i}⊗A_{c}^{i}⊗A_{s}^{i}\right)$$

Here, id represents the identity mapping branch.

The parallel fusion allows the network to combine directional and identity-path information according to learned channel and spatial weights. Horizontal or vertical features can contribute more strongly for elongated regions, whereas the identity path retains locally compact information. The ablation results in Section 4.4 evaluate the net effect of ASAM, but no claim is made that individual branch weights provide a causal explanation of landslide morphology.

### 3.3. Channel-Spatial Joint Gate

In the standard U-Net architecture, skip connections are commonly used to concatenate high-resolution shallow spatial features from the encoder with high-level semantic features from the decoder. However, direct feature concatenation often introduces substantial background noise from shallow networks—such as bare ground, buildings, and other elements resembling landslide spectra—into the decoder, which interferes with the final mask prediction.

Attention U-Net introduced decoder-guided gating to suppress irrelevant encoder responses before skip fusion [32]. Its forward operations are summarized as follows:

(9) $$\psi =ReLU({Conv}_{1\times 1}\left(x\right)+{Conv}_{1\times 1}(g))$$

(10) $$\alpha =\sigma ({Conv}_{1\times 1}(\psi ))$$

(11) Output=x⨂α

where x represents the encoder feature and g denotes the gate signal from the decoder.

The conventional attention gate aligns encoder and decoder features with 1 × 1 projections and produces a spatial coefficient map used to filter the encoder features. This is effective for localization, but the shared spatial coefficient does not retain a distinct spatial weighting pattern for every feature channel. CSJG is designed to preserve the channel-specific weighting while limiting additional projections.

We therefore introduce the Channel-Spatial Joint Gate (CSJG), a decoder-guided gating module that retains channel-specific spatial responses with a compact alignment operation.

**Figure 6:**
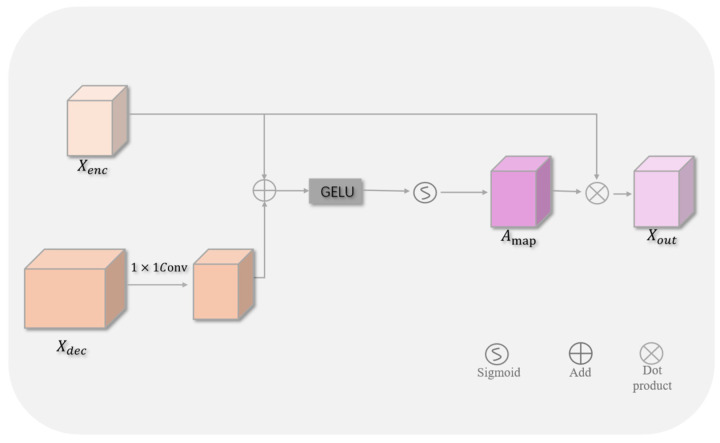
Channel-Spatial joint gate (CSJG). Decoder and encoder features are fused to generate a joint channel–spatial attention map, which reweights the encoder feature to produce the gated output.

As shown in Figure 6, we first perform a 1×1 convolution on the deep decoder feature $X_{dec}$ ($X_{dec}\in R^{N\times 2C\times H\times W}$), reducing its channel count C by half to align with the encoder feature $X_{enc}$ ($X_{enc}\in R^{N\times C\times H\times W}$). Then, we perform element-wise addition to achieve a preliminary aggregation of deep, strong semantics and shallow, high-resolution details.

(12) $$M=X_{enc}+{Conv}_{1\times 1}(X_{dec})$$

Here, $M\in R^{N\times C\times H\times W}$ is the fused intermediate feature map.

After obtaining the intermediate feature map M, unlike traditional Attention Gates, CSJG abandons channel compression operations and directly applies GELU and Sigmoid nonlinear mappings to generate the final attention feature map $A_{map}$. Finally, the generated attention feature map is multiplied element-wise with the original encoder feature $X_{enc}$ to filter out background noise.

(13) $$A_{map}=\sigma (GELU(M))$$

(14) $$X_{out}=A_{map}⊗X_{enc}$$

where σ represents the sigmoid activation function.

From Formula (13), it can be seen that the $A_{map}\in R^{N\times C\times H\times W}$ at this stage, which means that the generated mask retains independent and diverse weight distributions not only in the spatial dimensions but also across the channel dimension.

Compared to traditional attention gates, the entire CSJG module contains only a single 1×1 convolution, significantly reducing the number of model parameters. Moreover, the $A_{map}$ output by CSJG maintains the same number of channels as the input, allowing the network to allocate distinct spatial response distributions for each specific channel’s feature map. This design preserves channel-specific feature representations and allows the model to adaptively assign distinct spatial response patterns to different semantic features. This allows for more accurate identification of landslide areas amid complex background interference.

### 3.4. Design of PGD Loss Function

In landslide semantic segmentation, the number of foreground pixels corresponding to landslide regions is usually much smaller than the number of background pixels, resulting in a pronounced foreground–background class imbalance. Meanwhile, a large number of easily classified background pixels may dominate the training process, whereas pixels located near landslide boundaries or in regions spectrally similar to the surrounding background are generally more difficult to classify. When only a conventional pixel-wise loss is used, the model may become biased toward the dominant background class, thereby reducing its ability to identify minority landslide regions and ambiguous boundaries.

To address the issue of imbalanced positive and negative samples in segmentation, existing research often employs Focal Loss [16], which is defined as follows:

(15) $$L_{Focal}=-\frac{1}{N}{\left(1-p_{t,i}\right)}^{\gamma }\log\left(p_{t,i}\right)$$

where

(16) $$p_{t,i}=t_{i}p_{i}+(1-t_{i})(1-p_{i})$$

denotes the total number of pixels participating in the loss calculation, $p_{i}\in (0,1)$ is the predicted foreground probability of the i-th pixel, $t_{i}\in \{0,1\}$ is the corresponding ground-truth label, and γ is the focusing parameter.

As shown in Equation (15), Focal Loss reduces the weights of high-confidence, easily classified samples through the modulating factor ${\left(1-p_{t,i}\right)}^{\gamma }$. However, difficult pixels in landslide segmentation may arise from different factors, including boundary ambiguity, local texture degradation, and visual similarity between landslides and background objects such as bare soil, roads, and riverbanks. A single focusing parameter may not sufficiently reflect the differences among difficult pixels in the overall gradient distribution. Moreover, a relatively large γ may excessively suppress moderately difficult samples.

To mitigate the polarization of sample attention caused by the modulation factor γ and to simplify hyperparameter tuning, Poly Loss [22] is commonly employed in engineering as a replacement for Focal Loss. Poly Loss adjusts cross-entropy loss from the perspective of polynomial expansion. Its general form can be written as

(17) $$L_{Poly-J}=-\log\left(p_{t}\right)+\sum_{j=1}^{J}\epsilon_{j}{\left(1-p_{t}\right)}^{j}$$

where J denotes the maximum polynomial order, and $\epsilon_{j}$ is the adjustment coefficient of the j-th polynomial term. By introducing low-order probability terms, Poly Loss can flexibly adjust the optimization contributions of samples with different prediction confidences without requiring a complex modulating function.

To further alleviate the imbalance in the gradient distribution among pixels of different difficulty levels, this study combines a first-order probability regularization term with the Gradient Harmonizing Mechanism for Classification (GHM-C). This design is not intended as a theoretical reformulation of GHM-C. Instead, a first-order probability term is added to the gradient-density-based reweighting mechanism to increase the contribution of low-confidence pixels.

Let $z_{i}$ denote the network output of the i-th pixel before sigmoid activation. Its predicted foreground probability is

(18) $$p_{i}=\sigma (z_{i})$$

For a binary classification task using sigmoid activation and binary cross-entropy, the gradient magnitude of the i-th pixel is defined as

(19) $$g_{i}=\left|\frac{\partial L_{BCE,i}}{\partial z_{i}}\right|=\left|p_{i}-t_{i}\right|$$

During the calculation of the grouping weights, $g_{i}$ is detached from the computational graph so that the gradient-density statistics do not participate in backpropagation. Since $p_{i},t_{i}\in [0,1]$, the gradient magnitude satisfies $g_{i}\in [0,1]$.

The gradient range $\left[0,1\right]$ is then uniformly divided into $B_{g}$ intervals. The set of pixels belonging to the b-th gradient interval is defined as

(20) $${Ι}_{b}=\left\{i\left|e_{b}\leq g_{i}\leq e_{b+1}\right.\right\},\;\;\;\;\;b=1,2,\ldots ,B_{g}$$

${Ι}_{b}$ denotes the set of pixel indices i that satisfy $e_{b}\leq g_{i}<e_{b+1}$, where $e_{b}=\frac{b-1}{B_{g}}$, $e_{b-1}=\frac{b}{B_{g}}$.

To ensure that pixels with $g_{i}=1$ are assigned to the last interval, a small constant of $10^{-6}$ is added to the upper boundary of the final interval in the implementation.

The number of valid pixels in the b-th interval is

(21) $$n_{b}=\left|I_{b}\right|$$

and the number of non-empty gradient intervals is defined as

(22) $$K=\sum_{b=1}^{B_{g}}1(n_{b}>0)$$

where 1(⋅) denotes the indicator function. Take 1 if there are pixels in the b-th interval; otherwise, take 0.

Because momentum accumulation is not utilized in the current implementation, the weight of a pixel belonging to the b-th non-empty interval is given by

(23) $$\omega_{i}=\frac{N}{Kn_{b}},\;\;\;\;\;\;\;\;\;\;\;\;\;i\in I_{b}$$

According to Equation (23), pixels located in densely populated gradient intervals are assigned smaller weights, whereas pixels located in sparsely populated intervals receive larger weights. The GHM-C loss is therefore defined as

(24) $$L_{GHM-C}=\frac{1}{N}\sum_{i=1}^{N}\omega_{i}L_{BCE,i}$$

where the pixel-wise binary cross-entropy loss is

(25) $$L_{BCE,i}=-[t_{i}log(p_{i})+(1-t_{i})log(1-p_{i})]$$

By substituting Equation (23) into Equation (24), the GHM-C loss can equivalently be written as

(26) $$L_{GHM-C}=\frac{1}{K}\sum_{b=1}^{B_{g}}\frac{1}{n_{b}}\sum_{i\in I_{b}}L_{BCE,i}$$

From Equation (26), it is evident that each non-empty gradient interval contributes similarly to the final loss, thereby diminishing the dominant influence of high-density intervals containing a large number of easily classified pixels during the optimization process.

Based on GHM-C, this paper further introduces a first-order probability regularization term. According to Formula (16), when pixels are classified correctly with high confidence, $p_{t,i}$ approaches 1. Conversely, when the prediction confidence is low or misclassification occurs, $p_{t,i}$ is small. Therefore, the first-order probability regularization term is defined as follows:

(27) $$L_{PolyReg}=\frac{1}{N}\sum_{i=1}^{N}\left(1-p_{t,i}\right)$$

By combining the GHM-C loss with a first-order probability regularization term, this paper defines the Poly-Harmonized GHM loss as follows:

(28) $$L_{PolyGHM}=L_{GHM-C}+\epsilon L_{PolyReg}$$

where ϵ controls the contribution of the first-order probability term. In this study, it is set to

ϵ=2.0

It should be noted that in the implementation of this article, GHM-C is first calculated as a scalar loss, then added to the first-order probability regularization terms of all pixels and averaged.

To ensure stable global pixel-level supervision, this paper also retains the standard binary cross-entropy loss, which is defined as follows:

(29) $$L_{BCE}=-\frac{1}{N}\sum_{i=1}^{N}\left[t_{i}log(p_{i})+(1-t_{i})log(1-p_{i})\right]$$

In addition to pixel-wise supervision, Dice Loss is introduced to improve the regional overlap between the predicted landslide mask and the corresponding ground-truth mask. Let a mini-batch contain M images, with each image containing P  pixels. The Dice coefficient of the m-th image is defined as

(30) $$D_{m}=\frac{2\sum_{j=1}^{P}p_{m,j}t_{m,j}+\delta }{\sum_{j=1}^{P}p_{m,j}+\sum_{j=1}^{P}t_{m,j}+\delta }$$

where $p_{m,j}$ and $t_{m,j}$ denote the predicted foreground probability and ground-truth label of the j-th pixel in the m-th image, respectively, and δ is a smoothing constant.

The Dice Loss is calculated separately for each image and then averaged over the mini-batch:

(31) $$L_{Dice}=1-\frac{1}{M}\sum_{m=1}^{M}D_{m}$$

Finally, by combining the stable pixel-wise supervision of BCE Loss, the region-overlap constraint of Dice Loss, and the joint adjustment of gradient density and prediction confidence provided by PolyGHM Loss, the proposed Poly-Harmonized Gradient Dice Loss, abbreviated as PGD Loss, is defined as

(32) $$L_{PGD}=L_{BCE}+L_{Dice}+\mu L_{PolyGHM}$$

where μ controls the relative contribution of the PolyGHM component to the final composite loss. Based on the experimental results, μ is set to

 μ=0.5

The calculation process of PGD loss is shown in Algorithm 1.
**Algorithm 1.** Computation of PGD Loss.Input: logits Z; binary mask Y; B = 10; ε = 2.0; μ = 0.5; δ = 10^−5^ Output: scalar loss L_PGD 1: Compute P = sigmoid(Z) and detached gradient magnitude g = |stop-gradient(P) − Y|. 2: Partition [0, 1 + 10^−6^] into B bins and count K non-empty bins. 3: For each pixel i in a non-empty bin b containing n_b pixels, assign w_i = N/(K n_b). 4: Compute weighted BCE L_GHM and p_t = YP + (1 − Y)(1 − P). 5: Compute L_PolyGHM = L_GHM + ε·mean(1 − p_t), ordinary BCE, and image-wise Dice loss with smoothing δ. 6: Return L_PGD = L_BCE + L_Dice + μL_PolyGHM.

Detailed experiments can be found in Section 4.

### 3.5. Activation Function

In the feature learning process of neural networks, the nonlinear transformations introduced by activation functions directly influence the model’s ability to extract complex features. The original U-Net model employs the ReLU activation function, which is defined by the following formula:

(33) $$ReLU\left(x\right)=max(0,x)$$

As shown in Equation (33), ReLU sets negative inputs to zero. This supports efficient optimization but can suppress weak negative responses completely. We therefore use SiLU, whose smooth transition around zero retains non-zero gradients for negative inputs and may support more stable feature learning in low-contrast RGB regions. Its calculation is as follows:

(34) $$SiLU\left(x\right)=x\cdot \sigma \left(x\right)$$

where σ denotes the sigmoid function. Compared with ReLU, SiLU is smooth and non-monotonic near zero and retains small negative outputs. The activation-function ablation in Section 4.4 quantifies its empirical effect in the present architecture.

## 4. Experiment and Analysis

Our experiments used a merged RGB optical landslide dataset constructed from the public Moxi Town [37] and Bijie [38] image–mask collections. All 15 component-ablation configurations in Section 4.4 used the fixed random seed 42. We then compared AS-UNet with representative convolutional, attention-based, and Transformer-based segmentation models using seeds 42, 2026, and 77.

### 4.1. Experimental Setup and Reproducibility

Table 1 summarizes the computing environment. All experiments were run with PyTorch 2.3.0 and CUDA 11.8 on an NVIDIA GeForce RTX 4060 Laptop GPU and an Intel Core i7-12650H CPU; OpenCV-Python 4.12.0.88 was used for image processing.

Unless otherwise specified, all compared models used the common protocol in Table 2: SGD optimization for 150 epochs, an initial learning rate of 1 × 10^−3^, a minimum learning rate of 1 × 10^−5^, weight decay of 1 × 10^−4^, momentum of 0.9, a batch size of 16, CosineAnnealingLR scheduling, and early-stopping patience of two epochs. The same three random seeds (42, 2026, and 77) were used for the repeated model comparisons; input resolutions are reported in Section 4.5. Swin-UNet was initialized from swin_tiny_patch4_window7_224.pth and TransUNet from imagenet21k_R50+ViT-B_16.npz; all other compared models were trained without pretrained initialization. For reproducibility, the released implementation specifies encoder widths [32, 64, 128, 256, 512], bilinear upsampling with align_corners = True, 3 × 7 and 7 × 3 depthwise ASAM kernels, CSJG at the 256- and 128-channel skip connections, and deterministic cuDNN execution.

### 4.2. Dataset Construction and Splitting

The merged dataset contains 2565 RGB optical image–mask pairs: 1795 from Moxi Town and 770 from Bijie. Moxi Town images are provided as 512 × 512 pixels, whereas the Bijie collection contains mixed pixel dimensions. These pixel dimensions should not be interpreted as ground-sampling distances; consistent physical spatial-resolution metadata are not available across the two public sources. Only RGB images and binary masks were used, and any DEM content associated with the Bijie source was excluded. Each source was stratified into training, validation, and test subsets at a 7:2:1 ratio, yielding 1795, 513, and 257 images, respectively (Table 3). Because scene identifiers and uniform geolocation metadata are not available for all source images, cross-scene leakage and cross-region generalization cannot be independently audited from the supplied metadata and are treated as limitations.

The source-wise split and sample counts are reported in Table 3.

**Figure 7:**
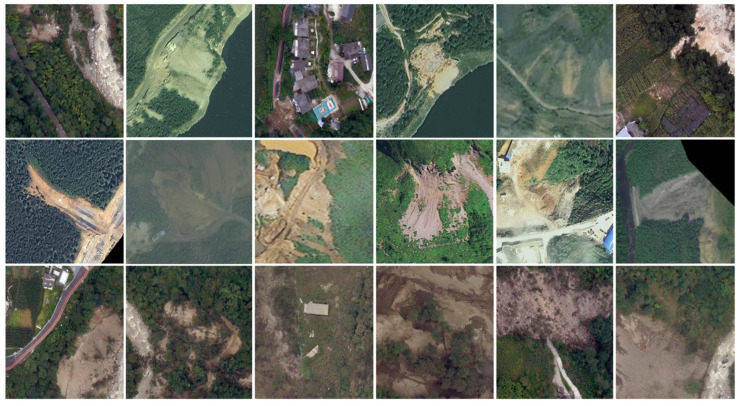
Representative RGB optical landslide images from the merged dataset.

Representative RGB images from the merged dataset are shown in Figure 7.

The dataset contains heterogeneous landslide shapes and backgrounds, including vegetation, river channels, bare soil, roads, and built-up areas. The evaluated segmentation setting therefore presents four recurring difficulties:

(1) Landslide pixels occupy a variable and often small fraction of each image, producing foreground–background imbalance.
(2) Landslide morphology varies from compact patches to elongated regions aligned with terrain features.
(3) Some image–mask pairs contain weak local texture or ambiguous boundaries, and resizing to the network input can further reduce boundary detail.
(4) Bare soil, riverbanks, shadows, and disturbed vegetation may resemble landslide material and cause false-positive predictions.

Training augmentation consisted of random 90° rotations, horizontal or vertical flips, and one of two photometric transformations: hue–saturation adjustment or brightness–contrast adjustment with equal selection probability. Images were resized to the model input size and normalized. Validation and test images underwent resizing and normalization only, without stochastic augmentation.

### 4.3. Evaluation Metrics

During the model evaluation process, the following four types of samples will be generated:

(1) TP (True Positive): Represents the pixels labeled as landslides and correctly detected by the model as landslides.
(2) TN (True Negative): Refers to pixels labeled as non-landslides and correctly identified by the model as non-landslides.
(3) FP (False Positive): Refers to pixels labeled as non-landslides but detected as landslides by the model, representing false positives.
(4) FN (False Negative) refers to pixels that are labeled as landslides but are detected by the model as non-landslides, representing missed detections.

Intersection over Union (IoU) was the primary segmentation metric. IoU measures the overlap between the predicted and reference landslide masks relative to their union and is defined as follows:

(35) $$IoU=\frac{TP}{TP+FP+FN}=\frac{Intersection}{Union}$$

where the intersection is the number of pixels shared by the predicted and reference foreground masks, and the union is the number of foreground pixels present in either mask.

Precision, recall, and F1 score were additionally reported to separate false-positive and false-negative behavior:

(1) Precision: Refers to the proportion of all samples identified by the model as landslides that are correctly labeled as landslide samples. A higher precision indicates that the model is more accurate and reliable in classifying pixels. The formula for precision is as follows:

(36) $$Precision=\frac{TP}{TP+FP}$$

(2) Recall: Refers to the proportion of samples labeled as landslides that the model correctly identifies as landslides. A higher recall rate indicates that the model more comprehensively recognizes landslides. The formula for calculating the recall rate is as follows:

(37) $$Recall=\frac{TP}{TP+FN}$$

(3) F1 Score: This metric considers both the precision and recall of the model and is defined as the harmonic mean of these two values. The formula for its calculation is as follows:

(38) $$F_{1}=\frac{2\times Precision\times Recall}{Precision+Recall}$$

### 4.4. Component Ablation and Robustness

To assess the impact of the loss function and modules used in this study on the U-Net model, 15 ablation experiments were conducted. The experimental setup and results are presented in Table 4.

Table 4 compares SiLU, PGD Loss, ASAM, and CSJG under the same fixed seed. Relative to the U-Net baseline (No.1; IoU = 0.7502), ASAM alone increased IoU to 0.7559, whereas ASAM + CSJG reached 0.7614. The combined gain is consistent with complementary roles for directional bottleneck context and channel-specific skip-connection gating.

Replacing ReLU with SiLU increased IoU from 0.7502 to 0.7635. PGD Loss produced the largest single-component gain, reaching 0.7724 with ReLU and 0.7904 with SiLU. Adding ASAM to the SiLU + PGD configuration increased IoU to 0.8009 (No.14), and the complete configuration with CSJG reached 0.8043 (No.15). All 15 configurations used seed 42; Table 5 extends the final comparison to three seeds.

To assess the consistency of the complete configuration, No.14 and No.15 were rerun with seeds 42, 2026, and 77 (Table 5). Adding CSJG increased mean IoU from 0.7995 ± 0.0039 to 0.8046 ± 0.0003, a gain of 0.0050, while reducing the run-to-run standard deviation from 0.0039 to 0.0003. The CSJG-equipped complete model therefore combined the highest mean IoU with the lowest run-to-run dispersion across the three seeds.

**Figure 8:**
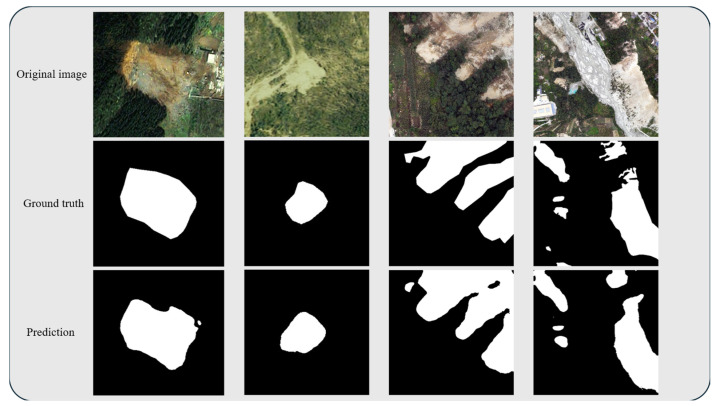
Representative segmentation results of the complete AS-UNet configuration. Rows show RGB inputs, reference masks, and predicted masks.

Figure 8 shows representative input images, reference masks, and predictions from the complete configuration.

### 4.5. Comparative Performance and Efficiency

We compared AS-UNet with U-Net, U-Net++, Attention U-Net, DeepLabV3+, TransUNet, and Swin-UNet. Accuracy was evaluated on the validation and independent test subsets, while FPS, FLOPs, parameter count, and peak memory were measured in the common hardware environment. The AS-UNet 128 × 128 configuration is the primary model; A 224 × 224 AS-UNet variant was set up specifically to match the input resolution of Swin-UNet, and this variant is used for direct comparisons between the two models.

Table 6 reports the mean ± sample standard deviation across seeds 42, 2026, and 77 for the best validation IoU attained in each run and the supplied independent-test IoU. Table 7 provides the corresponding validation precision, recall, and F1 score and records pretrained-weight usage.

Table 7 shows that Swin-UNet achieved the highest validation precision, recall, and F1 score; AS-UNet therefore does not dominate every metric. The direct comparison is made at the common input resolution of 224 × 224. Under this matched setting, AS-UNet achieved 81.09 ± 0.25% best validation IoU and 78.54 ± 0.27% test IoU, exceeding Swin-UNet by 1.57 and 1.51 percentage points, respectively. AS-UNet used 8.634 M parameters and 175.45 MB peak memory, which were 68.2% and 53.3% lower than Swin-UNet, and reached 255.63 FPS, 2.29 times the Swin-UNet throughput. This measured throughput advantage was retained despite the higher nominal FLOP count of AS-UNet at this resolution (22.066 G versus 11.822 G). Separately, the primary 128 × 128 AS-UNet configuration achieved 80.46 ± 0.03% validation IoU and 77.82 ± 0.32% test IoU with 7.206 G FLOPs, 166.30 MB peak memory, and 380.79 FPS. Thus, the resolution-matched comparison supports higher IoU together with lower parameter and memory requirements, while the 128 × 128 configuration provides the lower-cost deployment setting.

**Figure 9:**
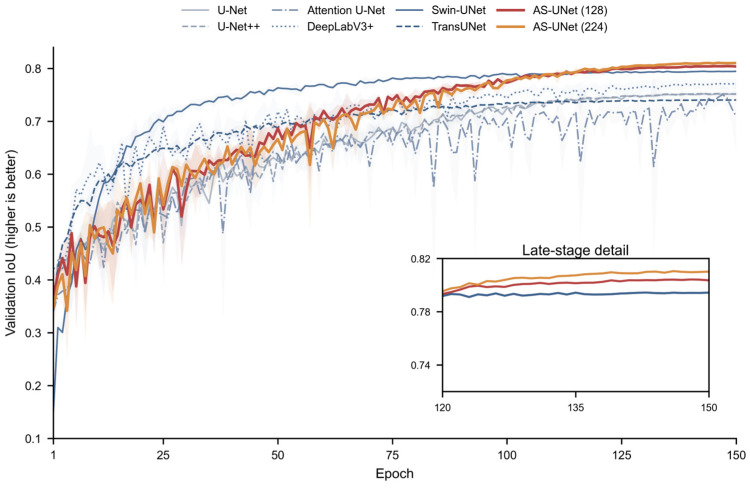
Validation-IoU trajectories across three random seeds. Lines show means and shaded bands show sample standard deviations; the inset magnifies epochs 120–150.

Figure 9 shows that AS-UNet converged more slowly than Swin-UNet early in training but continued to improve during later epochs. The three-seed bands narrow near convergence for both AS-UNet configurations. Attention U-Net shows larger late-stage variability because one run deteriorated after reaching its best checkpoint.

**Figure 10:**
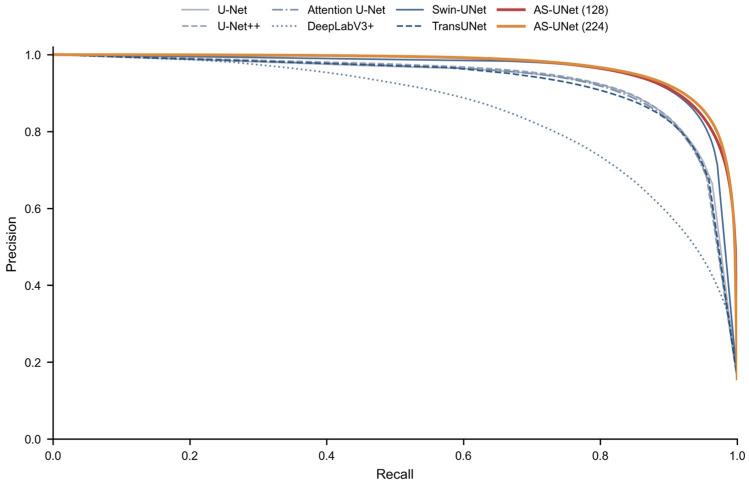
Representative precision-recall curves of the compared segmentation models.

Figure 10 shows that both AS-UNet configurations generally retain higher precision over most of the evaluated recall range than the included baselines, with the 224 × 224 variant forming the strongest overall profile and the 128 × 128 variant following closely. These representative threshold-level curves complement the three-seed IoU comparison in Table 6 and Figure 9.

### 4.6. Sensitivity and Loss-Function Comparisons

**Figure 11:**
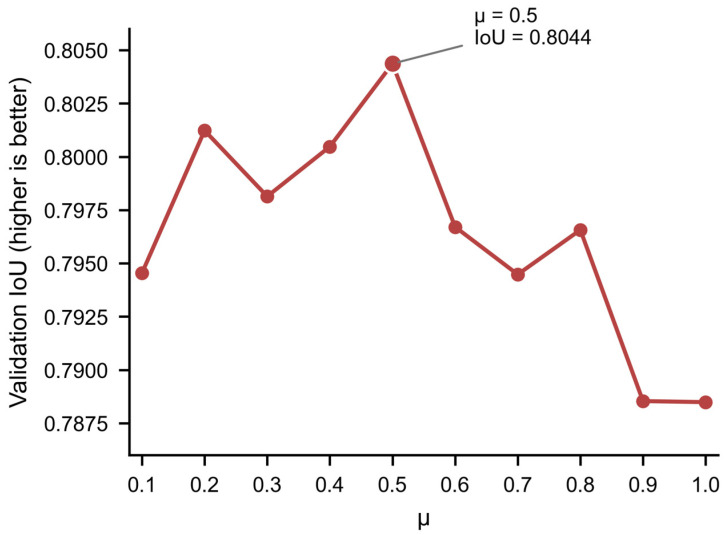
Sensitivity of validation IoU to μ.

To assess the sensitivity of PGD Loss to the weighting coefficient, validation IoU was evaluated for μ values from 0.1 to 1.0 (Figure 11).

The highest reported value, 0.8044, occurred at μ = 0.5. Performance declined for μ ≥ 0.6 and fell below 0.79 at μ = 0.9 and 1.0, indicating that excessive weighting of the corresponding loss component is unfavorable in the present dataset. This is an empirical setting for the reported protocol rather than a universal optimum.

**Figure 12:**
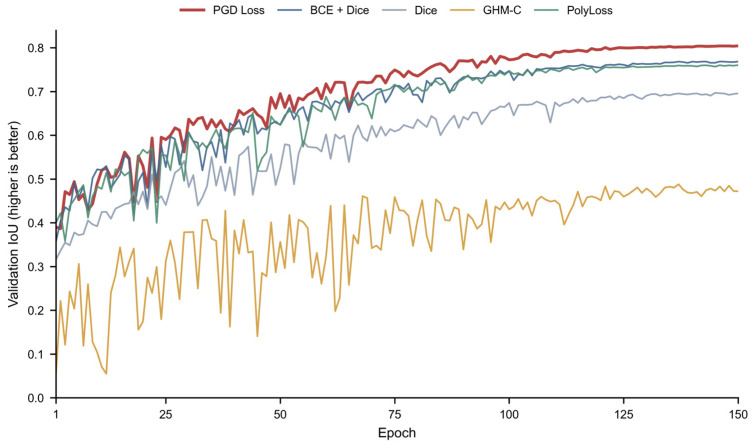
Representative validation-IoU trajectories for PGD Loss and alternative objectives.

In the representative run shown in Figure 12, PGD Loss reached a final validation IoU of 0.8044, compared with 0.7684 for BCE + Dice, 0.7603 for PolyLoss, 0.6957 for Dice Loss, and 0.4720 for GHM-C. The plot supports improved convergence under this run.

Overall, AS-UNet achieved a favorable balance between accuracy and efficiency on the evaluated RGB optical dataset. PGD Loss and SiLU produced the largest gains in the seed-42 ablation, and ASAM added a further improvement. In the repeated comparison, the complete model with CSJG achieved the highest mean IoU and the lowest run-to-run dispersion. Cross-dataset, cross-region, and multimodal generalization were not evaluated.

## 5. Conclusions

This study presents AS-UNet, a lightweight U-Net variant for binary landslide segmentation in RGB optical remote sensing imagery. ASAM introduces horizontal and vertical depthwise strip branches with joint channel-spatial reweighting; CSJG filters selected skip connections while retaining channel-specific spatial responses; and PGD Loss addresses class imbalance and ambiguous pixels through complementary pixel, region, gradient-density, and probability terms. Across three seeds, the 128 × 128 model achieved 80.46 ± 0.03% best validation IoU and 77.82 ± 0.32% independent-test IoU with 8.634 M parameters, 7.206 G FLOPs, and 380.79 FPS in the reported environment.

The evaluation has several limitations. The two public RGB sources lack harmonized ground-sampling-distance and scene-identity metadata. We also did not perform a cross-dataset holdout, k-fold cross-validation, SAR experiments, or multimodal fusion. Nevertheless, performance was consistent across the three reported seeds. Future work should examine scene-grouped k-fold cross-validation, scene-level data separation, cross-region validation, optical-SAR-DEM fusion, and deployment on edge hardware.

(1) Multi-source validation and fusion: Once reliable scene identifiers are available, perform scene-grouped k-fold cross-validation alongside source-held-out and geographically separated testing; then assess whether DEM, SAR, or multispectral inputs improve performance under explicitly modality-specific preprocessing.
(2) Lightweight and edge deployment: Quantify latency, energy use, and memory on UAV or embedded hardware and investigate pruning or quantization when the accuracy cost is acceptable.
(3) Label-efficient learning: Evaluate semi-supervised or weakly supervised strategies using unlabeled optical imagery while maintaining an independent, scene-disjoint test protocol.

## Figures and Tables

**Table 1:** Experimental environment and hardware configuration.

Environment and Configurations	Parameters
GPU	NVIDIA GeForce RTX 4060 Laptop
CPU	12th Gen Intel(R) Core(TM) i7-12650H (2.30 GHz)
Deep Learning Toolkit	pytorch2.3.0+cuda11.8
Computer Vision Toolkit	OpenCV-Python4.12.0.88

**Table 2:** Common network-training configuration.

Environment and Configurations	Parameters
Initial Learning Rate	0.001
Minimum Learning Rate	0.00001
Weight Decay	0.0001
momentum	0.9
batch_size	16
Epochs	150
optimizer	SGD
patience	2
scheduler	CosineAnnealingLR

**Table 3:** Source-wise dataset split.

Dataset	Modality	Image Size	Total	Train	Validation	Test
Moxi Town	RGB optical	512 × 512	1795	1256	359	180
Bijie	RGB optical	mixed	770	539	154	77
Total	RGB optical	mixed	2565	1795	513	257

**Table 4:** Component-ablation results with the fixed random seed 42.

Ablations					Metrics					
Groups	SiLU	PGDLoss	ASAM	CSJG	Params	FLOPs	Precision	Recall	F1 Score	IoU
No. 1					7.853 M	7.050 G	0.8539	0.8594	0.8551	0.7502
No. 2			√		8.469 M	7.063 G	0.8587	0.8617	0.8590	0.7559
No. 3				√	8.017 M	7.193 G	0.8549	0.8551	0.8537	0.7480
No. 4			√	√	8.634 M	7.206 G	0.8584	0.8682	0.8625	0.7614
No. 5	√				7.853 M	7.050 G	0.8670	0.8632	0.8638	0.7635
No. 6	√		√		8.469 M	7.063 G	0.8727	0.8588	0.8643	0.7643
No. 7	√			√	8.017 M	7.193 G	0.8726	0.8560	0.8632	0.7622
No. 8	√		√	√	8.634 M	7.206 G	0.8806	0.8563	0.8672	0.7688
No. 9		√			7.853 M	7.050 G	0.8728	0.8683	0.8695	0.7724
No. 10		√	√		8.469 M	7.063 G	0.8766	0.8824	0.8788	0.7870
No. 11		√		√	8.017 M	7.193 G	0.8725	0.8704	0.8703	0.7740
No. 12		√	√	√	8.634 M	7.206 G	0.8833	0.8765	0.8791	0.7875
No. 13	√	√			7.853 M	7.050 G	0.8904	0.8734	0.8810	0.7904
No. 14	√	√	√		8.469 M	7.063 G	0.8918	0.8845	0.8876	0.8009
No. 15	√	√	√	√	8.634 M	7.206 G	0.8983	0.8827	0.8896	0.8043

Note: All 15 configurations used the fixed random seed 42. √ indicates that a component is included. Runs 1–4 use ReLU with BCE + Dice; runs 5–8 add SiLU; runs 9–12 use PGD Loss with ReLU; and runs 13–15 combine PGD Loss with SiLU. ASAM is placed at the bottleneck, and CSJG is applied to the third and fourth skip connections.

**Table 5:** Three-seed performance and stability of the complete CSJG-equipped configuration.

Variant	CSJG	Precision	Recall	F1 Score	IoU
No. 14	–	0.8914 ± 0.0072	0.8831 ± 0.0029	0.8866 ± 0.0025	0.7995 ± 0.0039
No. 15	√	0.8933 ± 0.0046	0.8877 ± 0.0046	0.8898 ± 0.0001	0.8046 ± 0.0003

Note: Metric values are mean ± sample SD across seeds 42, 2026, and 77.

**Table 6:** Three-seed comparative performance and computational efficiency.

Model	Input	FPS	FLOPs (G)	Params (M)	Peak Memory (MB)	Val IoU (%)	Test IoU (%)
U-Net [11]	128 × 128	493.04	7.050	7.853	50.79	75.24 ± 0.20	72.08 ± 0.64
U-Net++ [39]	128 × 128	291.50	17.452	9.163	76.30	75.30 ± 0.47	72.86 ± 0.72
Attention U-Net [32]	128 × 128	350.45	7.304	7.985	167.22	75.91 ± 0.83	72.95 ± 0.76
DeepLabV3+ [40]	128 × 128	224.54	2.194	5.221	149.29	77.18 ± 0.91	75.58 ± 0.62
TransUNet [41]	128 × 128	70.90	16.104	93.231	540.52	74.08 ± 0.08	71.74 ± 0.51
Swin-UNet [29]	224 × 224	111.81	11.822	27.146	375.65	79.52 ± 0.25	77.03 ± 0.56
AS-UNet	128 × 128	380.79	7.206	8.634	166.30	80.46 ± 0.03	77.82 ± 0.32
AS-UNet	224 × 224	255.63	22.066	8.634	175.45	81.09 ± 0.25	78.54 ± 0.27

Note: IoU values are the mean ± sample SD across seeds 42, 2026, and 77. Validation values are the maximum IoU attained within each 150-epoch run; test values are from the independent test subset. Efficiency was measured in the hardware environment in Table 1 at the listed input size. Swin-UNet used swin_tiny_patch4_window7_224.pth and TransUNet used imagenet21k_R50+ViT-B_16.npz; all other models used no pretrained initialization.

**Table 7:** Three-seed validation precision, recall, and F1 score at the best-IoU checkpoint.

Model	Input	Pretrained Weights	Precision (%)	Recall (%)	F1 Score (%)
U-Net [11]	128 × 128	No	86.10 ± 0.65	85.47 ± 0.42	85.66 ± 0.13
U-Net++ [39]	128 × 128	No	86.55 ± 0.61	85.11 ± 1.02	85.69 ± 0.32
Attention U-Net [32]	128 × 128	No	86.65 ± 0.75	84.97 ± 1.17	85.75 ± 0.88
DeepLabV3+ [40]	128 × 128	No	79.49 ± 2.04	74.27 ± 0.20	76.79 ± 1.04
TransUNet [41]	128 × 128	Yes	84.36 ± 0.12	85.65 ± 0.16	84.81 ± 0.06
Swin-UNet [29]	224 × 224	Yes	91.58 ± 0.22	89.71 ± 0.29	90.63 ± 0.18
AS-UNet	128 × 128	No	89.33 ± 0.46	88.77 ± 0.46	88.98 ± 0.01
AS-UNet	224 × 224	No	89.78 ± 0.34	89.14 ± 0.25	89.39 ± 0.16

## Data Availability

The Moxi Town and Bijie source datasets are publicly available at the locations cited in References [37,38]. The AS-UNet implementation, trained model, split files, and released training/validation/test image—mask pairs are available at https://github.com/Youhaoran1512/AS-UNet (accessed on 14 July 2026).
